# Intracavitary Irradiation as a Safe Alternative for Cystic Craniopharyngiomas: Case Report and Review of the Literature

**DOI:** 10.1155/2016/3601395

**Published:** 2016-06-05

**Authors:** Alejandro Enriquez-Marulanda, Melibea Sierra-Ruiz, Luz Maritza Pabón, Javier Lobato-Polo

**Affiliations:** ^1^Fundación Clínica Valle del Lili, Cali, Colombia; ^2^Centro de Investigaciones Clínicas, Cali, Colombia; ^3^Universidad ICESI, Cali, Colombia

## Abstract

Craniopharyngioma treatment remains a challenge for clinicians and patients. There are many treatment alternatives; however one of them (intracavitary irradiation) seeks to control this type of benign brain tumor using minimally invasive techniques, with the specific aim of avoiding causing significant damage to important structures surrounding the sellar/suprasellar region. We present the case of a 3-year-old patient with a predominantly cystic craniopharyngioma who underwent intracavitary irradiation by stereotactic placement. Using this approach, the patient showed a successful response with remission of headaches and hydrocephalus. A reduction in the size of the cyst was achieved, without deterioration of visual fields, with no hormonal supplementation being needed, and with no evidence of focal neurological signs.

## 1. Introduction

Craniopharyngiomas are solid or mixed (cystic-solid) epithelial tumors originating from remnants of Rathke's pouch of the nasopharynx. It is estimated that in the United States there are 350 new cases of craniopharyngiomas diagnosed per year. These lesions are responsible of 1.2–4% of all childhood intracranial tumors and are considered to be the most common pituitary masses in this age group [1]. Despite their benign histological nature, these tumors should be considered as low grade malignancies, because, without treatment, they frequently reduce life expectancy; patients with such tumors have 3–6 times higher mortality than the general population and, overall, there is five-year survival rate of 80% [2, 3].

Such tumors affect child development, generating damage to the visual, endocrinologic, metabolic and neurocognitive functions, with such effects being more pronounced in children than in adults [2, 4]. Treatment remains a major challenge, because of the proximity of such tumors to important structures and also due to the high recurrence rates after partial resection. Treatment must find a balance between tumor control and the posttreatment quality of life in these patients, especially because this disease affects, disproportionately, the younger age group [4]. Here we present a case report of intracavitary treatment of a craniopharyngioma with radioactive isotopes which was performed with the specific aim of reducing the morbidity associated with aggressive surgical treatment.

## 2. Case Report Presentation

A 3-year-old female patient presented to the emergency room with a progressive headache localized in the right parietal region, which frequently woke her up at night and which was associated with diurnal somnolence and apparent right palpebral ptosis. She did not have any relevant antecedents in her past medical history and her developmental milestones were adequate for her age; she was, at the time, in kindergarten with a good general performance. Her general examination revealed that her weight (13 kg) was appropriate and other findings were unremarkable. In the neurological examination, there was evidence of right eye lateral rectus muscle discrete palsy and the fundoscopy revealed retinal venous ingurgitation.

**Figure 1:**
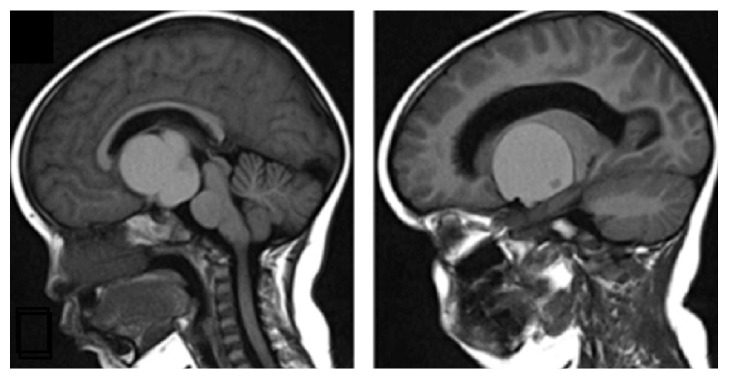
Pretreatment image. Initial Cerebral T1 weighted Magnetic Resonance Imaging showing large cystic suprasellar lesion.

A Magnetic Resonance Imaging (MRI) was performed and revealed a cystic suprasellar lesion of 5.31 × 3.9 × 3.9 cm with an additional nodule of 0.7 cm within the cyst. This lesion was located in the hypothalamic region and was compressing the optic chiasm, the right thalamus and basal ganglia structures, and the third ventricle, generating obstructive hydrocephalus. It also produced a compressive effect to the posterior side of the brainstem, occupying and broadening the interpeduncular cisterna. These findings suggested a cystic craniopharyngioma (Figure 1).

Due to the cyst size and the compression of adjacent structures, surgical management was indicated as a priority. Due to the high risk of postsurgical functional complications, however, we decided to treat the patient using interstitial irradiation. At first, the cyst was drained by aspiration via an Ommaya system placed with stereotactic guidance. This system had been cut in the distal portion to reduce the number of holes in the catheter, preventing leakage of the cyst substance. After the surgery, the patient and her parents noted that the severity of some of her symptoms decreased.

**Figure 2:**
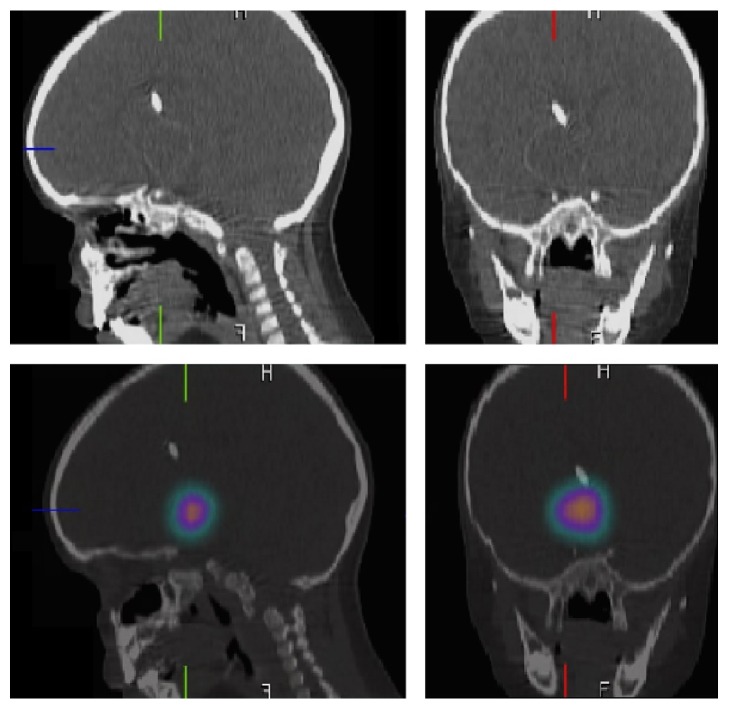
Postinjection Bremsstrahlung imaging by brain Single-Photon Emission Computed Tomography (SPECT). Brain SPECT coregistered with a computed tomography, showing a focal area of intense uptake in the suprasellar region, matching the shape of the cyst.

Four months later, injection of phosphorus-32 (^32^P) via the Ommaya reservoir system was performed, with the amount of ^32^P being calculated according to the cyst wall dose. The cyst size at time of instillation was 2.95 × 3.15 × 3.33 cm. A cyst volume of 15.5 mL was determined and then 2.5 mL of fluid from the craniopharyngioma was removed. Shortly after, 0.5 mL of colloidal ^32^P, 1.5 mL of technetium, and 0.5 mL of saline were injected in to the cyst. The instilled activity was equivalent to 2.8 mCi (0.5 mL). This quantity corresponds to 77.7 Megabecquerel of total radiation released. The resulting dose at the cyst wall was 200 Gy. A brain Single-Photon Emission Computed Tomography (SPECT) was performed immediately after the injection showing adequate placement of the isotopes (Figure 2). AnazaoHealth Corporation was the supplier of the colloidal ^32^P; this was imported to our country because we currently do not produce our radioisotopes.

**Figure 3:**
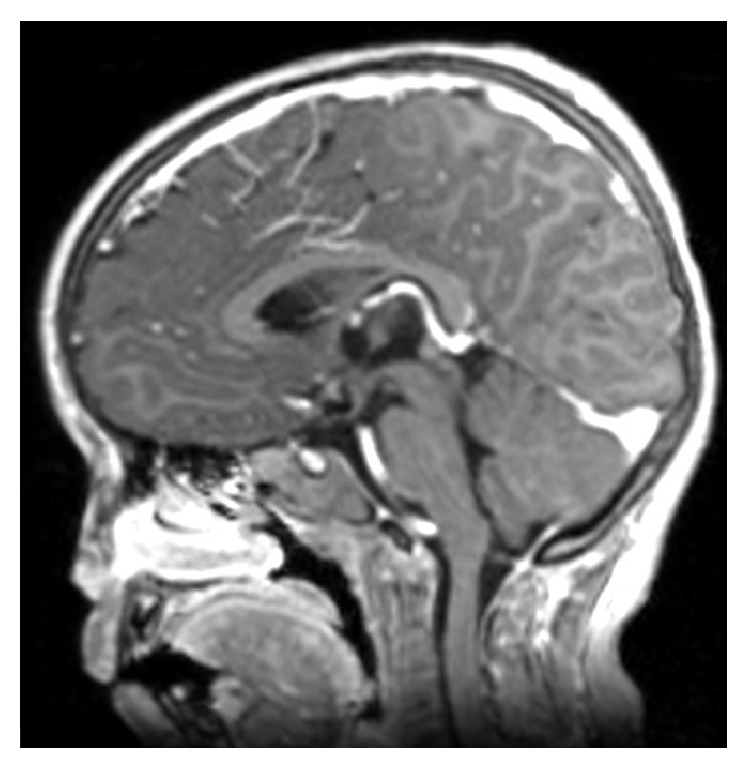
Follow-up image. Cerebral Magnetic Resonance Imaging enhanced with gadolinium contrast 22 months after intracavitary irradiation with radioisotope ^32^P.

The last MRI (Figure 3) was performed 21 months afterwards and showed a marked decrease of the signal intensity of the cyst content with evident size reduction. Also noted was a disappearance of the paracentral left nodular component and improvement of the hydrocephalus, as compared to the evidence from the patient's previous MRI. The last clinical follow-up was 22 months after treatment and showed that the patient was in an adequate condition with no recurring headaches. Additionally, she continued to perform well at her kindergarten and there were no sleep or behaviour disruptions. Her general examination revealed that her weight (16.8 kg) was appropriate and other findings were unremarkable. In the neurological examination, the patient did not exhibit any focal neurological symptoms, her extraocular movements were remarkably normal without palpebral ptosis, and there were no campimetry defects, evidencing a general improvement. Her endocrine and metabolic panel follow-up laboratories were TSH: 3.72 mUI/mL, FT4: 1.48 ng/dL, Cortisone: 20.28 *μ*g/dL, sodium: 139 mmol/L, Potassium: 4.09 mmol/L, and Chloride: 97.8 mmol/L; all were, therefore, within normal ranges with no hormonal supplementation required.

## 3. Discussion

Craniopharyngiomas treatment has various options; however there are two main alternatives that are typically used: one is an aggressive gross surgical removal leading to a complete resection, and the other is more conservative, using radiation therapy when a partial resection has been made or when the lesion has recurred.

For many years, complete surgical resection has been the first-line treatment; however this more aggressive method is associated with an unacceptably high risk of mortality and morbidity. Within the risks reported are hypothalamic damage, visual impairment, and endocrine complications, which range between 45 and 90%, with associated manifestations such as anterior hypopituitarism, insipid diabetes, growth alterations, and feeding and behavioural abnormalities [5]. If a partial resection is used, disease recurrence ranges between 50 and 91% of cases [6]. As such, both of these techniques represent poor options for treatment.

Due to the high morbidity and mortality associated with the aggressive surgical approach, the trend in treatment nowadays is to control the tumor without causing significant damage to important structures surrounding the sellar/suprasellar region. This is why minimally invasive interventions such as ionizing radiation-based techniques have gained popularity and seem to represent a promising strategy for treatment: they allow improved precision, reducing long-term toxicity by limiting the exposure of surrounding healthy tissue to ionizing radiation [4, 6–8].

The case presented is an example of the success of intracavitary irradiation. The purpose of this procedure was to reduce the size of the cyst in order to relieve compression on the third ventricle and other structures, such as the visual pathway and hypothalamus, without generating unacceptable damage to them. We preferred instead the Ommaya system of direct puncture and injection, as we considered that this would minimize the probability of radioisotope leakage through the puncture site made by the stereotactic needle. A SPECT showed adequate placement of the radioisotope and did not show any leakage of the ^32^P outside the target cyst (Figure 2). This is called Bremsstrahlung imaging which detects the photons emitted by beta radiation emitting radioisotopes as they lose energy in the body. It is important, however, to note that the Bremsstrahlung imaging is not sensitive enough to exclude leakage of the radioisotope, image quality is very poor, and often anatomical landmarks are not visible [9].

The intracavitary irradiation approach is achieved using beta radiation emitting radioisotopes such as ^90^Y, ^186^Re, and ^32^P applied into the cyst using stereotactic or neuroendoscopic approaches [4, 10, 11]. The therapeutic range is only a few millimeters, allowing the destruction of the cyst lining secretory epithelium and stopping fluid production, thus subsequently reducing the cyst size and promoting the adhesion of the cyst wall [4, 12]. This can be achieved because radioisotopes allow delivery of higher doses of radiation directly into the inner surface of the cyst without affecting the surrounding brain structures; this is in contrast to the results obtained using conventional external beam radiotherapy techniques [6, 12]. The radiation dose that can be administered by beta radiation emitting radioisotopes to the cyst is between 90 and 300 Gy [13–16]. Craniopharyngiomas exhibit a cystic composition in approximately 90% of cases. The literature shows that the mean response and control rates of craniopharyngioma with predominant cyst composition using intracavitary irradiation are between 67 and 88%, resulting in decreased cyst size, and even the disappearance of the lesion, with a 10-year survival probability ranging from 61 to 80% [4, 16–23]. Unfortunately, this data comes from observational studies and there are no randomized clinical trials available at this point [4]. For treatment with intracavitary irradiation, it is important to know the ratio between the cystic and solid part of the tumor, since tumors with a solid composition are insufficiently controlled compared to those with high cystic content [6, 10, 11]. In the particular case presented, we found that the solid part of the tumor disappeared, due to the effects of the radiation: the patient will, however, be monitored and surgical resection will be performed should there be any recurrence of the solid part.

Despite being a less aggressive approach, this treatment is not free from adverse effects to the surrounding tissue. In the literature reviewed, there were a few cases where complete blindness or worsening of the visual fields has occurred; cases of diabetes insipidus, panhypopituitarism, third nerve palsy, and injury of the internal carotid artery have also been reported [4, 6, 7]. Another important thing to mention is that, despite necessary precautions during the application of the radioisotope, leakage of an intracystic substance remains a potential risk [4]. The final problem with this technique is the possibility of recurrence, especially when there is a concomitant solid part. Despite these negative aspects of the treatment, favorable results are far more frequent than unfavorable results. The radioactivity dose does, however, need to be carefully selected, in order to achieve a safe yet effective treatment, reducing the risk of radiation accidents [4, 12].

## 4. Conclusion

Intracavitary irradiation using radioisotopes in patients with craniopharyngiomas of predominantly cystic composition may be an effective, tolerable, and relatively safe option for the treatment of these lesions either as a primary approach or following other, unsuccessful, therapies.

## Abbreviations

MRI: Magnetic Resonance Imaging
^90^Y: Yttrium-90
^186^Re: Rhenium-186
^32^P: Phosphorus-32
SPECT: Single-Photon Emission Computed Tomography.
